# The Frequency-Dependent Aerobic Exercise Effects of Hypothalamic GABAergic Expression and Cardiovascular Functions in Aged Rats

**DOI:** 10.3389/fnagi.2017.00212

**Published:** 2017-06-30

**Authors:** Yan Li, Ziqi Zhao, Jiajia Cai, Boya Gu, Yuanyuan Lv, Li Zhao

**Affiliations:** ^1^Department of Exercise Physiology, Beijing Sport UniversityBeijing, China; ^2^College of Life Sciences, University of Chinese Academy of SciencesBeijing, China; ^3^Key Laboratory of Physical Fitness and Exercise, Ministry of Education, Beijing Sport UniversityBeijing, China

**Keywords:** aerobic exercise, aging process, cardiovascular function, PVN, GABAergic neurons

## Abstract

A decline in cardiovascular modulation is a feature of the normal aging process and associated with cardiovascular diseases (CVDs) such as hypertension and stroke. Exercise training is known to promote cardiovascular adaptation in young animals and positive effects on motor and cognitive capabilities, as well as on brain plasticity for all ages in mice. Here, we examine the question of whether aerobic exercise interventions may impact the GABAergic neurons of the paraventricular nucleus (PVN) in aged rats which have been observed to have a decline in cardiovascular integration function. In the present study, young (2 months) and old (24 months) male Wistar rats were divided into young control (YC), old sedentary, old low frequency exercise (20 m/min, 60 min/day, 3 days/week, 12 weeks) and old high frequency exercise (20 m/min, 60 min/day, 5 days/week, 12 weeks). Exercise training indexes were obtained, including resting heart rate (HR), blood pressure (BP), plasma norepinephrine (NE), and heart weight (HW)-to-body weight (BW) ratios. The brain was removed and processed according to the immunofluorescence staining and western blot used to analyze the GABAergic terminal density, the proteins of GAD67, GABA_A_ receptor and gephyrin in the PVN. There were significant changes in aged rats compared with those in the YC. Twelve weeks aerobic exercise training has volume-dependent ameliorated effects on cardiovascular parameters, autonomic nervous activities and GABAergic system functions. These data suggest that the density of GABAergic declines in the PVN is associated with imbalance in autonomic nervous activities in normal aging. Additionally, aerobic exercise can rescue aging-related an overactivity of the sympathetic nervous system and induces modifications the resting BP and HR to lower values via improving the GABAergic system in the PVN.

## Introduction

Aging is associated with many chronic disorders, which also is the dominant risk factor for cardiovascular diseases (CVDs), and accounts for more deaths than any other disease. Among these factors age is an independent risk factor and is also implicated in the development of hypertension, grouped under CVDs. Structure remolding and dysfunctional changes of the cardiovascular system can be induced by advanced aging, which contribute to an increased risk of hypertension (Ferrari et al., 2003; Lakatta and Levy, 2003; Najjar et al., 2005; North and Sinclair, 2012; Illi et al., 2015). The autonomic nervous system is critical in the normal function of heart rate (HR), blood pressure (BP), cardiac output and vascular structure. On the other hand, levels of sympathetic nerve activity (SNA) that are too high result in increased peripheral vascular resistance, remodeling of cardiovascular tissue and, ultimately, hypertension if these persist over a long period of time. Aging related increase in levels of baseline plasma norepinephrine (NE) concentration (Palmer et al., 1978; Sowers et al., 1983) and SNA and decline in parasympathetic nervous activity were suggested by experimental research which was performed on both human beings and animals (Rowe and Troen, 1980; Ebert et al., 1992; Esler et al., 1995; Lavi et al., 2007; Scridon et al., 2012). Numerous studies have shown that aging-related changes in autonomic nervous system functions are responsible for high BP in the elderly (Barnes et al., 2014; Hart and Charkoudian, 2014). An important question in this field is whether aging-induced neural changes in the cardiovascular regulation brain area is associated with aging-related dysfunctional changes in the autonomic nervous system.

The paraventricular nucleus (PVN) of the hypothalamus plays a major role in central cardiovascular and volume control, and has been implicated in controlling autonomic nerve activity, and involved in neuroendocrine function. Immunohistochemistry has shown that GABAergic, mainly inhibitory neurons in the CNS, account for 50% of cells in PVN. Electrophysiological studies have revealed that the vast majority of PVN inputs are GABAergic synapses. The GABAergic deficits have been shown in hypertension animal models (Martin and Haywood, 1998; Li and Pan, 2006). Correcting the impaired GABAergic system in the PVN can attenuate the development of high BP in spontaneously hypertensive rats (SHRs; Ye et al., 2012; Song et al., 2014). Thus, the GABAergic system in the PVN represents an excellent candidate for aging-related changes that can contribute to the alteration of BP modulation by the autonomic nervous system in aging persons.

Epidemiological studies have determined that regular physical activity, especially chronic aerobic exercise profoundly affects cardiovascular parameters. It is recommended to treat hypertension along with pharmaceutical antihypertensive therapies. Also exercise training appears to prevent and restore aging-related impairment in autonomic nervous regulation (Carter et al., 2003; De Meersman and Stein, 2007; Wichi et al., 2009; Mancia and Grassi, 2014), but the extra central mechanism is still unclear. Positive effects of exercise on motor and cognitive capabilities, as well as on brain plasticity has been reported for young (Madroñal et al., 2010) and aged (Gruart et al., 2008) mice, as well as for experimental models of Alzheimer disease (AD; García-Mesa et al., 2011). Recent studies suggest a link between exercise training and the GABAergic system in the PVN in normotensive rats and SHRs (Hsu et al., 2011; Jia et al., 2014). Although normal aging is associated with specific and relatively subtle excitatory synaptic alterations in the hippocampus and prefrontal cortex (Burke and Barnes, 2006; Mattson and Magnus, 2006), there is a shortage of studies in the GABAergic system of the PVN addressing the changes in the normal aging brain. Whether the GABAergic system in the hypothalamus of senile animals alters or plays a regulatory role in exercise-induced cardiovascular adaptation remains unknown.

In the current study, we used normal aging rats to examine the changes of the GABAergic system in the PVN and to determine whether aerobic exercise rescues aging related over activity of the sympathetic nervous system and induces modifications of the resting BP and HR. Two different exercise frequency paradigms were set to choose which one is more beneficial to aging animals. A noninvasive tail-cuff system was used to measure cardiovascular parameters in conscious animals. Autonomic nervous activities were quantified by spectral analysis of original BP and HR tracings and plasma NE level. The exercise effects on the hypothalamic protein levels of various GABAergic system markers were determined by immunoblotting, and the population percentage of hypothalamic GABAergic neurons was estimated by observation under a fluorescence microscope. The information obtained in this research will help to understand the central mechanisms of exercise-induced benefits on aging-related hypertension. The results will also contribute to understanding the underlying exercise-induced synaptic plasticity in aging brain.

## Materials and Methods

### Animals and Exercise Protocol

Twenty-four month old male Wistar rats were utilized as aging rats (Cichon et al., 2012) and 2 month old male Wistar rats were used as young controls (YCs). All rats were grouped into YC, old sedentary (O-SED), old low frequency exercise (O-LEX) and old high frequency exercise (O-HEX). Animals were maintained under temperature control (24 ± 5°C) and 12 h light-dark cycle (lights on between 07:00 and 19:00), and provided with standard chow and tap water *ad libitum*. All experimental protocols were approved by the ethical committee of Beijing Sport University and were performed in accordance with the Chinese animal protection laws and institutional guidelines. The experiment design was approved by the Beijing Sport University Science Experimental Ethics Committee, approval number 2015015. All studies involving animals are reported in accordance with the ARRIVE guidelines for reporting experiments involving animals (Kilkenny et al., 2010). Exercised animals were introduced to treadmill running for 5 days at 10 m/min, once a day for 10–20 min. In the 12 weeks training program (Koltai et al., 2012), exercised animals ran at 12 m/min, 0 slope, for 60 min/day. The running frequency is 3 days/week for O-LEX, whereas 5 days/week for O-HEX. Hsu’s study has used only an intensity of approximately 70% of maximal oxygen consumption for 60 min/day, 5 days/week for 8 weeks (Hsu et al., 2011). In our study, considering cardiorespiratory fitness and exercise capacity declined with aging, we choose two frequencies of forced exercise (3 times per week and 5 times per week) at the same treadmill intensity to determine which frequency was better for aged animals. Animals were used for cardiovascular parameter testing and then sacrificed 2 days after the last exercise session to avoid the metabolic effects of the final run.

### Measurement of Cardiovascular Parameters

The BP and HR of animals were measured using a noninvasive tail-cuff system (BP-300A, Chengdu TME Technology Co., Chengdu, China). The heart was weighed using an electronic balance. The heart weight/body weight (HW/BW) ratio was calculated.

### Power Spectral Analysis of BP Signals

Spectral analysis was performed offline on the original HR tracing using BP-300A software (Chengdu TME Technology Co., China) based on the manufacture’s protocol. Briefly, a 70 s stable segment was selected. Then, the spectral analysis function was run on the segment and the data was collected to calculate the mean value and SEM. The low-frequency band (0.27–0.74 Hz) and the high-frequency band (0.75–3.85 Hz) served as the marker for the sympathetic nervous activity and the parasympathetic nervous activity, respectively (Hsu et al., 2011; Wu et al., 2012; Chon et al., 2014).

### Measurement of Plasma Norepinephrine Level

Blood samples were collected using EDTA SST tubes (BD, Franklin Lakes, NJ, USA). After centrifugation at 3000 rpm at 4°C, the plasma was collected and stored at −20°C. The plasma NE level was detected using a rat NE ELISA kit (H096, Jiancheng, Nanjing, China).

### Immunofluorescent Staining

Rats were anesthetized with urethane and transcardially perfused with 4% paraformaldehyde in 0.1 M phosphate-buffered saline (PBS) pH 7.4. Brains were dissected out from the skulls, postfixed at 4°C in 4% paraformaldehyde for 48 h and then dehydrated with 30% sucrose in 0.1 M PBS at 4°C. Coronal sections (40 μm) were made on a cryostat (CM1850, Leica, Wetalar, Germany) according to the following coordinates (Li and Pan, 2007; bregma): 1.6–2.0 mm caudal, 1.8–2.2 mm lateral and 7.8–8.1 mm deep from the surface of the cortex. Then tissues were mounted using a drop of PBS. Sections were washed in PBS three times for 5 min. Antigen retrieval was done by heating sections in sodium citrate buffer (10 mM sodium citrate, 0.05% Tween 20, pH 6.0) at 95°C for 15 min. After washing in PBS, sections were blocked with 10% BSA (0218054980, MP Biomedicals, Santa Ana, CA, USA) for 30 min at room temperature. After blocking, sections were incubated overnight at 4°C in mouse monoclonal to GAD-67 antibody (1:200, ab26116, Abcam, Cambrige, UK). The next day, sections were washed in PBS three times for 5 min, and incubated with fluorescein (FITC)-conjugated affinipure goat anti-mouse IgG (H + L) (1:200, SA00003-1, Proteintech Group, Chicago, IL, USA) for 60 min in the dark at room temperature. After washing, sections were covered with antifade solution (070044-A, Cellchip Biotechnology, Beijing, China). Sections were visualized using a fluorescence microscope (ECLIPSE 50i, Nikon, Melville, NY, USA). The total number of GABAergic neurons were counted in the sections using the optical fractionator method.

### Protein Extraction and Western Blot Analysis

Immediately after animal sacrifice, the PVN was collected and kept frozen in liquid nitrogen. Tissues were homogenized with RIPA buffer (89900, Pierce, Thermo Fisher Scientific, Rockford, IL, USA) containing 25 mM Tris-HCl pH 7.6, 150 mM NaCl, 1% NP-40, 1% sodium deoxycholate, 0.1% SDS and protease inhibitors (04693159001, Roche, Mannheim, Germany). Lysates were then centrifuged at 13,000 *g* for 30 min at 4°C on the Centrifuge 5424R (Eppendorf, Hamburg, Germany) and the supernatants were collected. Protein concentration was determined using the BCA Protein Assay Kit (23227, Pierce) on the xMark™Microplate Absorbance Spectrophotometer (Bio-Rad Laboratories, Hercules, CA, USA). Samples were diluted in a 2X sample buffer containing 50 mM Tris-HCl pH 6.8, 2% SDS, 10% glycerol, 0.1% bromophenol blue and 5% β-mercaptoethanol and then heated at 100°C for 15 min. Twenty microgram total protein was electrophoresed on 10%–12% polyacrylamide gels in the Mini-PROTEAN Tetra Cell (165-8001, Bio-Rad) and subsequently transferred to PDVF membranes (IPVH00010, Millipore, Billerica, MA, USA). Membranes were then blocked with 5% BSA (0218054980, MP Biomidicals) in TBS containing 0.1% Tween 20 (0777, Amresco, Solon, Ohio) for 2 h at room temperature, incubated in fresh washing buffer for 10 min, and then incubated at 4°C with the following primary antibody: GAD-67 (1:1000, ab26116, Abcam), GABA_A_ receptor γ2 (Stuck et al., 2012; 1:1000, AB5559, Chemicon, Merck Millipore, Billerica, MA, USA), gephyrin (1:800, sc-25311, Santa Cruz Biotechnology, Dallas, TX, USA) and GAPDH (1:4000, 60004-1-Ig, Proteintech Group) overnight. After three washes in TBS-Tween 20, membranes were incubated for 1 h at room temperature in horseradish peroxidase-conjugated anti-rabbit or anti-mouse second antibody (111-035-003 or 115-035-003, Jackson ImmunoResearch Laboratories, West Grove, PA, USA). After repeated washing, membranes were developed using Supersignal West Pico (34080, Pierce), and protein bands were visualized on a ChemiDocTM XRS+ System (Bio-Rad). The bands were quantified by Image Lab software (Bio-Rad), and normalized to GAPDH, which served as an internal control.

### Statistical Analysis

Results are expressed as mean ± SEM, and *n* denotes the number of animals used in each experiment. Data sets were compared with one-way analysis of variance followed by Tukey *post hoc* test for multiple comparisons. Results were considered to be significant at *p* < 0.05.

## Results

### Effects of Aerobic Exercise Training on Age-Related Physical Characteristics

Aging related increase in the stiffness of vessels and BW have remarkable consequences for high BP. Normally, the functional ability of large conduit vessels to store energy during systole allow a systolic BP which is lower while the energy is stored in the tension of the vessels, and the ability to recoil to release that energy during diastole keeps the diastolic pressure high enough to push the blood through the capillaries throughout the cardiac cycle. With aging, the major conduit arteries stiffen, which reduces the ability to absorb some of the energy during systole. This causes systolic pressure to increase to above-normal levels. Concurrently, diastolic pressure falls below normal because there is less recoil of the elastic conduit vessels during diastole. This is not the only explanation for elevated SBP and lower DBP in elderly individuals, but it provides a conceptual framework for vessel changes over time. In order to investigate the effects of aerobic exercise on aging related changes in physical characteristics, we examined the BW, HW, HR and BP of all rats. As shown in Table 1, BW were higher in the O-SED group than in the YC group (*p* < 0.05). However, the O-SED group exhibited a lower HW/BW than in the YC group (*p* < 0.05). There was no significant difference in BW among the O-SED, O-LEX and O-HEX groups at the beginning of exercise. After aerobic exercise training for 12 weeks, BW was significantly lower in both O-LEX and O-HEX groups (*p* < 0.01 for O-LEX and *p* < 0.05 for O-HEX). Whereas there was no significant difference in HW among the O-SED, O-LEX and O-HEX group (*p* > 0.05). Therefore, the HW/BW ratio was markedly increased in the O-LEX and O-HEX groups (*p* > 0.05), and the HW/BW was higher in O-HEX than in O-LEX (*p* > 0.05). The HR and SBP in the O-SED group were higher than in the YC group (*p* < 0.01 for HR and *p* < 0.05 for SBP). Compared with the O-SED group, the HR and SBP in the O-LEX and O-HEX group were reduced (*p* < 0.05). The DBP in the O-SED group was lower than in the YC group (*p* < 0.01). Compared with the O-SED group, the DBP in the O-LEX and O-HEX group were increased (*p* < 0.05). Moreover, the HR and SBP in the O-HEX group were much lower than in the O-LEX group (*p* < 0.05). These results are consistent with findings that exercise induces a lower resting HR and BP in normal vessels of young animals (Hsu et al., 2011) and moreover suggest that in aged animals, aerobic exercise can enhance vascular compliance of aging conduit vessels to reverse higher SBP and HR, and lower DBP and HW/BW ratio. At the same time, even in aged animals, a higher frequency of moderate intensity is better to modulate cardiovascular function.

**Table 1:** Body weight, heart weight and blood pressure (BP) of rats after 12 weeks of aerobic exercise in each group.

	YC	O-SED	O-LEX	O-HEX
BW (g)	586 ± 12	694 ± 69*	602 ± 37^##^	623 ± 38^#^
HW (g)	1.7 ± 0.05	1.66 ± 0.12	1.59 ± 0.14	1.72 ± 0.16
HW/BW ratio (mg/g)	2.9 ± 0.1	2.4 ± 0.2*	2.6 ± 0.2^#^	2.8 ± 0.2^#&^
HR (beats/min)	450 ± 9	530 ± 6**	518 ± 2^#^	500 ± 7^#&^
SBP (mmHg)	115 ± 6.2	126 ± 3.5*	116 ± 7.7^#^	106 ± 9.6^#&^
DBP (mmHg)	101 ± 1.9	79.5 ± 4.0**	87.1 ± 0.4^#^	87.5 ± 6.6^#^

Values are mean ± SEM. BW, body weight; HW, heart weight; HW/BW ratio, heart weight/body weight ratio; HR, heart rate; SBP, systolic blood pressure; DBP, diastolic blood pressure. *p < 0.05 vs. YC; **p < 0.01 vs. YC; ^#^p < 0.05 vs. O-SED; ^##^p < 0.01 vs. O-SED; ^&^p < 0.05 vs. O-LEX. n = 12 in each group.

### Aerobic Exercise Training Regulated Aged-Related Changes in Corticosterone Level and the Neural Controls

**Figure 1:**
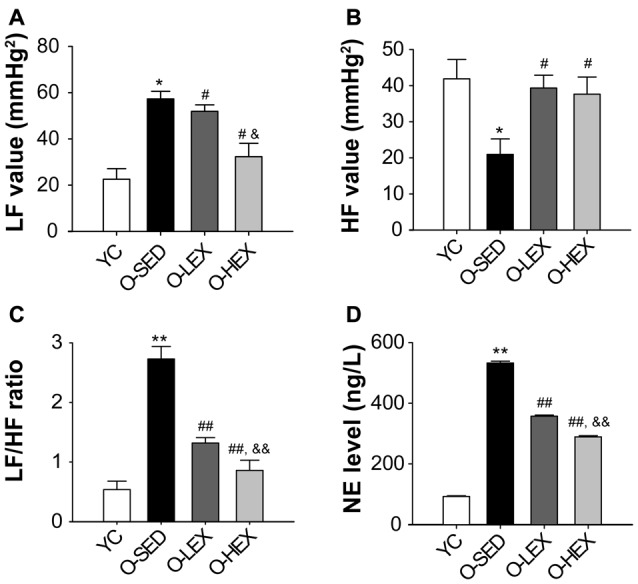
Effects of 12 weeks of aerobic exercise on autonomic nervous system modulation. **(A–C)** the LF value **(A)**, HF value **(B)** and LF/HF ratio **(C)** in young control (YC), old sedentary (O-SED), old low frequency exercise (O-LEX) and old high frequency exercise (O-HEX) group, respectively. **(D)** The plasma NE level in each group. *p < 0.05 vs. YC; ^#^*p* < 0.05 vs. O-SED; ^&^*p* < 0.05 vs. O-LEX; **p < 0.01 vs. YC; ^##^*p* < 0.01 vs. O-SED; ^&&^*p* < 0.01 vs. O-LEX. *n* = 12 in each group.

With aging, one factor influencing the stiffening large vessels is an increase in sympathetic tone in smooth muscle. Many studies have shown that the plasma NE level provides a good indicator of sympathetic nervous activity (Lake et al., 1976; Kawada et al., 2014). In order to delineate the effect of different exercise volumes on the sympathetic nervous activity of old rats, we tested the plasma NE level by ELISA. The NE level in the plasma of the rats was detected 48 h after final running to avoid the effect of the final exercise section on the NE concentration in the plasma. As shown in Figure 1, the plasma NE level was markedly increased in the O-SED group, compared with the YC group (*p* < 0.01). The plasma NE level was decreased by aerobic exercise (*p* < 0.01), and larger training volume had a larger effect on it (*p* < 0.01).

**Figure 2:**
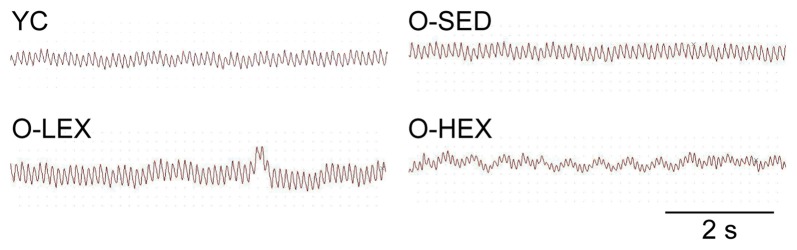
Representative heart rate (HR) traces for each group. HR and blood pressure (BP) of animals from each group were measured using a BP-300A noninvasive tail-cuff system. The HR trace was used for a spectral analysis with BP-300A software. YC, young control group; O-SED, old sedentary group; O-LEX, old low frequency exercise group; O-HEX, old high frequency exercise group. Bar = 2 s.

Also to examine the possibility that increased NE level is associated with sympathetic activity, we examined the autonomic neural control by analyzing pulse wave power spectrum. The contributions of sympathetic and parasympathetic controls to the regulation of HR and BP were tested by calculation of the power spectral density of the HR tracings. Original HR traces (pulse wave) in resting for each group are shown in Figure 2. As shown in Figure 1, compared with the YC group, the O-SED group has a higher LF value and a lower HF value (*p* < 0.05), therefore the LF/HF ratio is increased in the O-SED group (*p* < 0.01). Regular aerobic exercise training (both 3 days/week and 5 days/week) decreased the LF value and increased the HF value (*p* < 0.05), therefore the LF/HF ratio in the O-LEX and O-HEX groups is decreased, compared with the O-SED group (*p* < 0.01). Moreover, the LF value and the LF/HF ratio in the O-HEX group were lower than in the O-LEX group (*p* < 0.05 for LF value and *p* < 0.01 for LF/HF ratio). Hsu et al. (2011) have reported that chronic moderate exercise resulted in lower mean BP due to lowering sympathetic and elevating parasympathetic pressure in young rats. Based on our observation of the exercise on the sympathetic tone in old rats, this suggests that not only in young animals, but in aged animals, aerobic exercise of moderate intensity can suppress the age-related elevated sympathetic outflow to modulate BP.

### Effects of Aerobic Exercise Training on Aged-Related Changes in Endogenous GABAergic Activities of Paraventricular Nucleus

Numerous studies mainly of hippocampus and cortex have shown that aged-related neuron loss has been associated with impairments in brain function (Stanley et al., 2012). A reduced number of GABAergic interneurons in the aged brain result in an increased excitability of hippocampal pyramidal cells. But little material has reported age-related changes in inhibitory interneurons in the PVN of the hypothalamus. Thus, we examined the density of interneurons positive for the GABA-synthesizing enzyme glutamate decarboxylase-67 (GAD-67) in the PVN of the hypothalamus to evaluate the center control of SNA in old rats.

**Figure 3:**
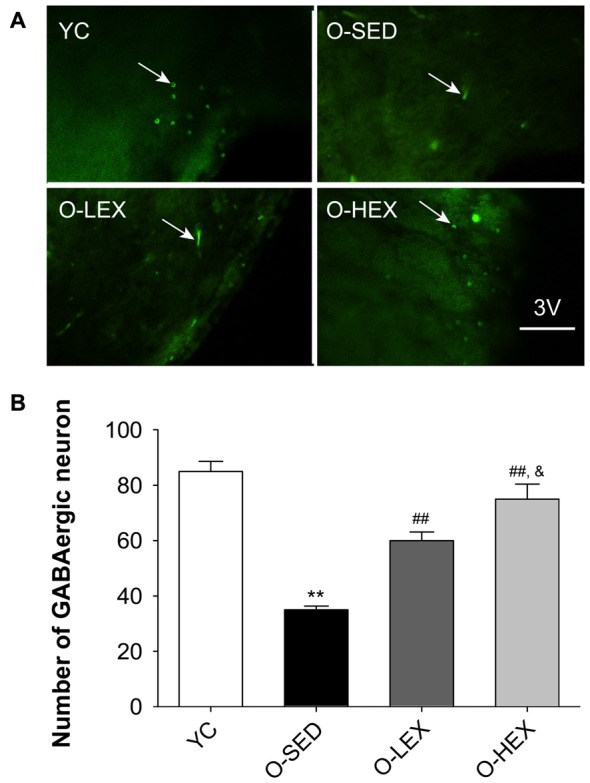
Effects of 12 weeks of aerobic exercise on the number of GABAergic neurons in the paraventricular nucleus (PVN). **(A)** Immunofluorescence staining of GABAergic neurons (GAD67-positive) in the PVN in YC, O-SED, O-LEX and O-HEX group, respectively. **(B)** Number of GABAergic neurons in the PVN in YC, O-SED, O-LEX and O-HEX group. 3V, the third ventricle. Scale bar = 50 μm. **p < 0.01 vs. YC; ^##^*p* < 0.01 vs. O-SED; ^&^*p* < 0.05 vs. O-LEX. *n* = 7 in each group.

Figure 3A shows the GAD-67 positive neurons in the PVN of the hypothalamus. Figure 3B shows the number of GABAergic neurons in the PVN of the four groups. The total number of the GABAergic neurons was less in the O-SED group than in the YC group (*p* < 0.01), whereas regular aerobic exercise training (both 3 days/week and 5 days/week) increased the immunoreactivity of the GAD-67 positive neuronal density (*p* < 0.01). Furthermore, the O-HEX group had more GABAergic density in the PVN than the O-LEX group (*p* < 0.05).

**Figure 4:**
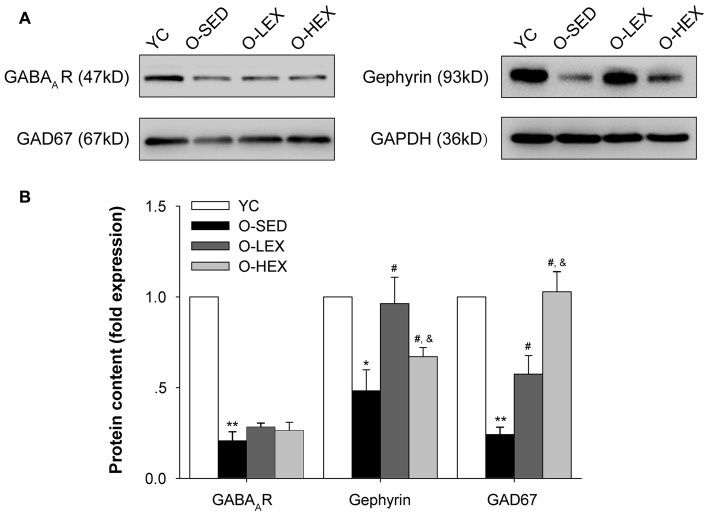
Effects of 12 weeks of aerobic exercise on the GABAergic related protein expressions in the PVN. **(A)** Immunoreactive bands corresponding to GABA_A_ receptor, gepryrin, GAD67 and GAPDH in the PVN in YC, O-SED, O-LEX and O-HEX group, respectively. **(B)** Data collections of GABA_A_ receptor, gepryrin and GAD67 protein levels as a ratio to GAPDH. *p < 0.05 vs. YC; **p < 0.01 vs. YC; ^#^*p* < 0.05 vs. O-SED; ^&^*p* < 0.05 vs. O-LEX. *n* = 7 in each group.

Protein expressions of GAD-67, GABA_A_ receptor and gephyrin are shown in Figure 4. Compared with the YC group, there was significantly less expression of GAD-67 (*p* < 0.01), GABA_A_ receptor (*p* < 0.01) and gephyrin (*p* < 0.05) protein in the O-SED group. In addition, aerobic exercise training (both 3 days/week and 5 days/week) was associated with significant increases in the expression of GAD-67 and gephyrin protein (*p* < 0.05). Protein expression of GAD-67 in the O-HEX group was significantly higher than in the O-LEX group (*p* < 0.05). However, there was less expression of gephyrin protein in the O-HEX group than in the O-LEX group (*p* < 0.05). As for GABA_A_ receptor, there was no statistically significant difference among O-SED, O-LEX and O-HEX groups (*p* > 0.05).

These results show a significant decrease in GABAergic neurons in the hypothalamus of old rats (24 months), suggesting a decline in autonomic regulation due to inhibitory neuron declines with aged compared to YC (2 months). Similar to Hsu’s findings that 8 weeks of moderate exercise elevated the GABAergic system in the hypothalamus in 4–5 weeks, in very young rats (Hsu et al., 2011), we observed an increase of GABAergic neurons in the PVN of 24 months old rats which experienced 12 weeks of aerobic exercise training, indicating that exercise-induced neuroplasticity can occur at any age.

## Discussion

The potential positive effects of exercise within the brain are of particular interest in the context of neuronal plasticity (Gruart et al., 2008; Madroñal et al., 2010; García-Mesa et al., 2011). In agreement, our data show that aged rats have a poorer proportion of GABAergic neurons expressing GAD-67 in the PVN. Additionally, the present study extends the existing literature by demonstrating that aerobic exercise particularly in aged animals, increases GABAergic expression and regulates autonomic nervous system activity as evidenced by changes in cardiovascular parameters at rest. That is, with increasing age, resting BP and HR appeared higher than those in the Young group, and exercise training in old rats augmented the GABAergic system (higher activity, more synaptic terminals and upregulated protein levels) in the PVN of the hypothalamus. This led to lower resting BP and HR, suppressing the sympathetic tone and to enhancing parasympathetic activity under conscious conditions. The effects of exercise training on the GABAergic system of the PVN appeared to be affected in a “dosage”-dependent way, a high frequency was better than a low frequency. Moreover, the PVN has neuron projections to the medullar vasomotor center and peripheral cardiovascular tissues, GABAergic adaptations in the hypothalamus should play an important role in mediating the beneficial effects of exercise training on the cardiovascular regulation.

First, we determined that regular exercise training induced adaptive changes in cardiovascular integration function in aging animals. Compared with the O-SED group, the animals that performed 12-week aerobic exercise had a lower BW, but the HW/BW ratio was markedly increased. Moreover, a significant increase in baseline SBP appeared in the aged rats. As we know, in healthy sedentary adults, aging is associated with increased stiffness (reduced compliance) of large elastic arteries (London and Guérin, 1999). The results here indicate that the aging animals did have common aging features such as a higher SBP and rest HR, while a lower DBP (stiffening of large arteries). After 12 weeks of regular aerobic exercise training, rats in the O-LEX and O-HEX groups showed a decreased HR and SBP.

Aging is an important risk factor of hypertension (Higashi et al., 2012). The autonomic nervous system plays an important role in both the acute and chronic regulation of BP and the development of hypertension in humans and animal models (Mancia and Grassi, 2014). Accumulating evidence shows that with age, the balance between the sympathetic nervous activity and the parasympathetic nervous activity changes. The autonomic imbalance contributes to increased HR, cardiac output and total peripheral vascular resistance resulting in hypertension (Barnes et al., 2014). It has been shown that regular aerobic exercise exerts its protective and therapeutic effects on hypertension by regulating the autonomic nervous system (Pagani et al., 1988; Iwane et al., 2000; Masson et al., 2015). Here we performed power spectral analysis on HR traces to get a better characterization of the effect of different exercise volumes on the autonomic regulation. The data shows that the LF band of the power spectral density of HR traces was O-SED > O-LEX > O-HEX > YC, this is evidence that the activity of the sympathetic nerve increased in old rats and 12 weeks of treadmill exercise decreased age-related increase in the sympathetic nervous activity, which depended on the exercise volume. Also the HF band of the power spectral density of BP traces was O-SED < O-LEX = O-HEX < YC, indicating decreased activity of the parasympathetic nerves with aging, and aerobic exercise can improve the parasympathetic nervous activity in old rats. Consistent with the increase of activity in sympathetic nerve from the power spectral analysis, the plasma NE level was also increased in O-SED rats. These results indicated that during aging sympathetic nervous activity was more sensitive to exercise intervention than the parasympathetic nervous activity.

The hypothalamus is the cardiovascular integration center, where the PVN of the hypothalamus is an important autonomic nucleus which plays a key role in the initiation of endocrine and autonomic regulation of HR and BP (Zhang et al., 1997; Dampney et al., 2005; Erdos et al., 2015). The PVN can modify the sympathetic outflow by regulating the activities of the roatal ventrolateral medulla neurons and the parasympathetic outflow via the nucleus of solitary tract or the nucleus ambiguous (Coote, 2005; Chen et al., 2013). Accumulating evidence indicates that the GABAergic system in the PVN plays an important role in the regulation of BP. First, GABA, GAD-67 and GABA receptors are expressed in the PVN. Second, compared with normotensive rats, hypertension rats have lower levels of GABA and GAD-67. Third, GABAergic synaptic inputs in the PVN inhibit sympathetic outflow and BP. Impaired control of GABAergic neurons in the PVN may lead to the elevated sympathetic drive in hypertension. Forth, the function of the GABA_A_ receptor is attenuated in hypertensive animal models (Li and Pan, 2007; Cork et al., 2016). Finally, the effects of some endogenous neurotransmitters are mediated by GABA (Zhang and Patel, 1998; Li and Pan, 2005; Rojas-Piloni et al., 2007). It is likely that aerobic exercise can decrease BP via regulating the GABAergic system in the PVN in normotensive adult rats and SHRs (Hsu et al., 2011; Jia et al., 2014). The present study was undertaken to understand the hypothesis that an aging-related decrease in the GABAergic system function in the PVN contributes to aging-associated HR and BP changes. Regular exercise can improve the aging-related impaired GABAergic system in the PVN.

A reduction of GABAergic function in the PVN can be a result of either presynaptic or postsynaptic change. We hypothesized that the GABA release in the PVN should reduce in aging rats and can be attenuated by regular aerobic exercise. GABA is synthesized by GAD67 and released from GABAergic neurons in the PVN (Chattopadhyaya et al., 2007; Lau and Murthy, 2012). Thus, a decrease in GABAergic density or protein level of GAD67 may contribute to a reduction in GABA release. The results showed that advanced aging decreased the GABAergic density and the GAD67 protein level in the PVN. Both high and low frequency exercise increased the GABAergic density and enhanced the expression of GAD67. The increased effect was exercise volume dependent. These results suggest that the aging-associated HR and BP changes might be attributed to the reduction in GABA release in the PVN. There might be a volume-dependent increased effect of exercise on the presynaptic GABA release and this result is consistent with results found from normotensive rats and SHRs (Hsu et al., 2011; Jia et al., 2014). This might be the presynaptic mechanism in the PVN whereby exercise decreases BP in older people.

It has been shown that the attenuated function of GABA_A_ receptors in the PVN may contribute to the development of hypertension (Li and Pan, 2007). A Gruart et al. (2008) report that aged wild-type mice and AD models presented similar impairment in discrete motor learning such as delay eyeblink conditioning and deficits in synaptic plasticity independent of β-amyloid load, suggesting that people should pay more attention to the progressive decline of synaptic plasticity during normal aging. We found that the protein level of GABA_A_ receptor in the PVN decreased in aged rats and the protein level significantly increased after running exercise. In order to clarify the effects of aerobic exercise on the expression of GABA_A_ receptor on the post synaptic membrane and its function, we tested the protein level of gephyrin. Gephyrin is one of the most functional postsynaptic proteins in inhibitory synapses. It is believed to function as a scaffold at inhibitory synapses, analogous its function to that of PSD-95 at glutamatergic synapses. It can bring GABA_A_ receptors and stabilize them in the inhibitory synapses (Essrich et al., 1998; Kneussel et al., 1999; Jacob et al., 2005; Choii and Ko, 2015). Studies showed that the modulation of gephyrin was accompanied by changes in the clustering and function of GABA_A_ receptor (Petrini et al., 2014). Our results showed that exercise can attenuate the aging induced decreased expression of gephyrin, suggesting exercise can improve the clustering and function of GABA_A_ receptor. We also found that the low exercise volume group had a higher expression of greqhyrin than the high exercise volume group, indicating that the clustering and function of the GABA_A_ receptor in the PVN might be better in the low volume exercise group. Neuronal activity regulates the surface number and/or function of GABA_A_ receptors. Several lines of evidence suggest that chronic exposure to GABA may induce the GABA_A_ receptor endocytosis (Gutiérrez et al., 2014). In the present study, we found out that 5 days/week exercise may have more presynaptic GABA release than 3 days/week exercise group, so this might be one of the mechanisms underlying the less surface number of GABA_A_ receptors observed in the high frequency exercise group.

Exercise is regarded as a behavioral intervention to enhance brain health and plasticity (van Praag et al., 2005). Recent evidence indicates that experience stimuli (like motor learning) drove changes in the synaptic plasticity involved in associative memory retention and the experience-induced synaptic plasticity in the hippocampus might be attributed to the alternations of LTP-associated protein (especially PKMζ; Madroñal et al., 2010). Similarly, even under neuropathological conditions, such as AD, voluntary physical exercise impeded the cognitive deficits and improved sensorimotor function in the AD triple transgenic mouse model (3xTg-AD) through various physiological mechanisms, including reducing oxidative stress, maintaining redox homeostasis and up-regulating BDNF in the brain (García-Mesa et al., 2014). Our prior research has shown that exercise, especially regular aerobic exercise attenuated aging-associate synaptic changes via regulating the expression and activity Rho GTPases in cortex and hippocampus (Li et al., 2017). Here we showed that the aging-associated GABAergic impairment in the PVN can be restored by exercise. It provides strong evidence that exercise training-induced neural plasticity is also present in different brain areas and also retained at any age.

In conclusion, this study provides evidence for the effect of different volumes of exercise in aging rats on the GABAergic system of the PVN by a combination of functional, morphological and molecular methods, and suggests that in aging rats, regular aerobic exercise can modulate the aging related imbalance of autonomic nerve activity via improving the function and protein level of the GABAergic system in the PVN to decrease the HR and BP. The results also indicate that high frequency exercise has better effects on the aging induced changes in the GABAergic system in the PVN. Of course to understanding mechanisms underlying the benefit effects of exercise on aging-induced impaired regulation of cardiovascular system in the PVN, further investigations on the glutamatergic system are absolutely needed.

## Author Contributions

YLi and LZ contributed to the conception and design of the study. YLi, ZZ, JC, BG and YLv performed the experiments, analyzed the data and contributed to their interpretation. YLi, ZZ and LZ wrote the manuscript.

## Conflict of Interest Statement

The authors declare that the research was conducted in the absence of any commercial or financial relationships that could be construed as a potential conflict of interest.
